# Candidemia in Internal Medicine: Facing the New Challenge

**DOI:** 10.1007/s11046-022-00624-x

**Published:** 2022-03-17

**Authors:** Lucia Brescini, Sara Mazzanti, Gianluca Morroni, Francesco Pallotta, Annamaria Masucci, Elena Orsetti, Roberto Montalti, Francesco Barchiesi

**Affiliations:** 1 Dipartimento di Scienze Biomediche e Sanità Pubblica, Università Politecnica delle Marche, Ancona, Italy; 2 Clinica Malattie Infettive Azienda Ospedaliera Universitaria Ospedali Riuniti Umberto I°-Lancisi-Salesi, Ancona, Italy; 3 Laboratorio di Microbiologia, Azienda Ospedaliera Universitaria Ospedali Riuniti Umberto I°-Lancisi-Salesi, Ancona, Italy; 4 Azienda Ospedaliera Universitaria Ospedali Riuniti Umberto I°-Lancisi-Salesi, Ancona, Italy; 5 Malattie Infettive, Ospedale Murri, Fermo, Italy; 6 Unità di Chirurgia Epato-Bilio-Pancreatica, Mininvasiva e Robotica, Dipartimento di Sanità Pubblica, Università Federico II, Napoli, Italy; 7 Malattie Infettive, Azienda Ospedaliera Ospedali Riuniti Marche Nord, Pesaro, Italy

**Keywords:** Candidemia, *Candida albicans*, Internal medicine, Antifungal agents, Antifungal susceptibility testing

## Abstract

Candidemia is an alarming problem in critically ill patients including those admitted in Internal Medicine Wards (IMWs). Here, we analyzed all cases of candidemia in adult patients hospitalized over nine years (2010–2018) in IMWs of a 980-bedded University Hospital of Ancona, Italy. During the study period, 218/505 (43%) episodes of candidemia occurred in IMWs patients. The cumulative incidence was 2.5/1000 hospital admission and increased significantly over time (*p* = 0.013). Patients were predominantly male, with a median age of 68 years. Cardiovascular diseases and solid tumors were the most frequent comorbidities. *Candida albicans* accounted for 51% of the cases, followed by *C. parapsilosis* (25%), *C. tropicalis* (9%) and *C. glabrata* (7%). Thirty-day mortality was 28% and did not increased significantly over time. By multivariate logistic regression analysis, the presence of neutropenia (OR 7.247 [CI95% 1,368–38,400; *p* = 0.020]), pneumonia (OR 2.323 [CI95% 1,105–4,884; *p* = 0.026]), and being infected with *C. albicans* (OR 2.642 [95% CI 1,223–5,708; *p* = 0.013) emerged as independent predictors of mortality. The type of antifungal therapy did not influence the outcome. Overall, these data indicate that patients admitted to IMWs are increasingly at higher risk of developing candidemia. Mortality rate remains high and significantly associated with both microbiologic- and host-related factors.

## Introduction

*Candida* spp. is the major causative agent of fungal infections in hospitalized patients and the fourth most common cause of nosocomial bloodstream infection (BSI) [1, 2]. Candidemia, which is associated with significant morbidity and mortality, has been frequently reported in hematological, surgical and critical care patients [3, 4]. However, in the last years, the incidence of candidemia in Internal Medicine Wards (IMWs) greatly increased [5]. Literature data show that at least 1/3 of candidemic patients are hospitalized in IMWs [6]. These patients are usually old, they are affected by multiple comorbidies and their mortality rate is quite high [7–9].

For over 10 years we have started a candidemia surveillance program in our institution, a university hospital with nearly 1,000 beds. Here, we describe the incidence, demographics, clinical and microbiologic characteristics of patients with candidemia hospitalized in IMWs from 2010 to 2018.

## Patients and Methods

*Hospital setting, study design, data collection and definitions.* The setting is a 980-bedded University Hospital in Ancona, Italy including five intensive care units (ICUs), 11 medical and 11 surgical wards. All cases of BSIs due to *Candida* spp. between January 1, 2010 to December 31, 2018 were retrieved from the microbiology laboratory database and patients’ charts were reviewed retrospectively by three of the authors (LB, SM and EO). A case of *Candida* BSI was defined as a peripheral isolation of *Candida* spp. from blood culture in a patient with temporally related clinical signs and symptoms of infection. Appropriate antifungal therapy was considered when an appropriate drug (based on subsequent in vitro susceptibility testing results) with adequate dosage was started within 72 h from the first blood culture performed. Adequate dosage of an antifungal agent was defined according to IDSA 2009—2016 guidelines [10, 11]. Early central venous catheter (CVC) removal was defined a removal of the line within 48 h from drawing blood culture. A catheter-related candidemia was defined according to the guidelines of the Infectious Diseases Society of America [12]. Mortality was calculated at 30 days from the occurrence of the episode of *Candida* BSI. To ascertain the outcome, we considered only those patients from which clinical information was included in the regional health surveillance system. The present research was performed in accordance with the ethical standards of the 1964 Declaration of Helsinki and its later amendements. The Institutional Review Board of the Azienda Ospedaliero-Universitaria Ospedali Riuniti Umberto I°-Lancisi-Salesi granted retrospective access to the data without need for individual informed consent. The consent was not given since the data were analyzed anonymously.

*Microbiology.* Yeast isolates were recovered from blood samples using BacT/ALERT (bioMérieux) and identified with the MALDI-TOF Biotyper™ (Bruker Daltonics, Germany). Antifungal susceptibility testing of fluconazole, amphotericin B and caspofungin was performed using the SensitreYeastOne colorimetric plate (Trek Diagnostic System). The three drugs were selected since each of them is the representative of a specific class. MICs were interpreted according to latest species-specific clinical breakpoints (CBPs) as established by the Clinical and Laboratory Standards Institute (CLSI) [13].

### Statistical Analysis

Incidence of candidemia was calculated per 1000 hospital admissions using annual hospital activity. Linear regression analysis was utilized to define the correlations between years and incidence of candidemia and mortality. Categorical variables were expressed as absolute numbers and their relative frequencies; continuous variables were expressed as median and interquartile range (IQR). Categorical variables were compared by the χ2 or Fisher exact test, while continuous variables were evaluated by the Student *t* test (for normally distributed variables) or the Mann–Whitney *U* test (for nonnormally distributed variables). Variables which reached a statistical significance (*p* < 0.05) at univariate analysis were analyzed by multivariate logistic regression analysis to identify independent risk factors for 30-day mortality. Results were expressed as odds ratio and 95% CI. All statistical analyses were performed using the statistical package SPSS for Windows v. 20 (SPSS Inc., Chicago, IL, USA). A *p* value < 0.05 was considered to represent statistical significance and all statistical tests were two-tailed.

## Results

**Fig. 1:**
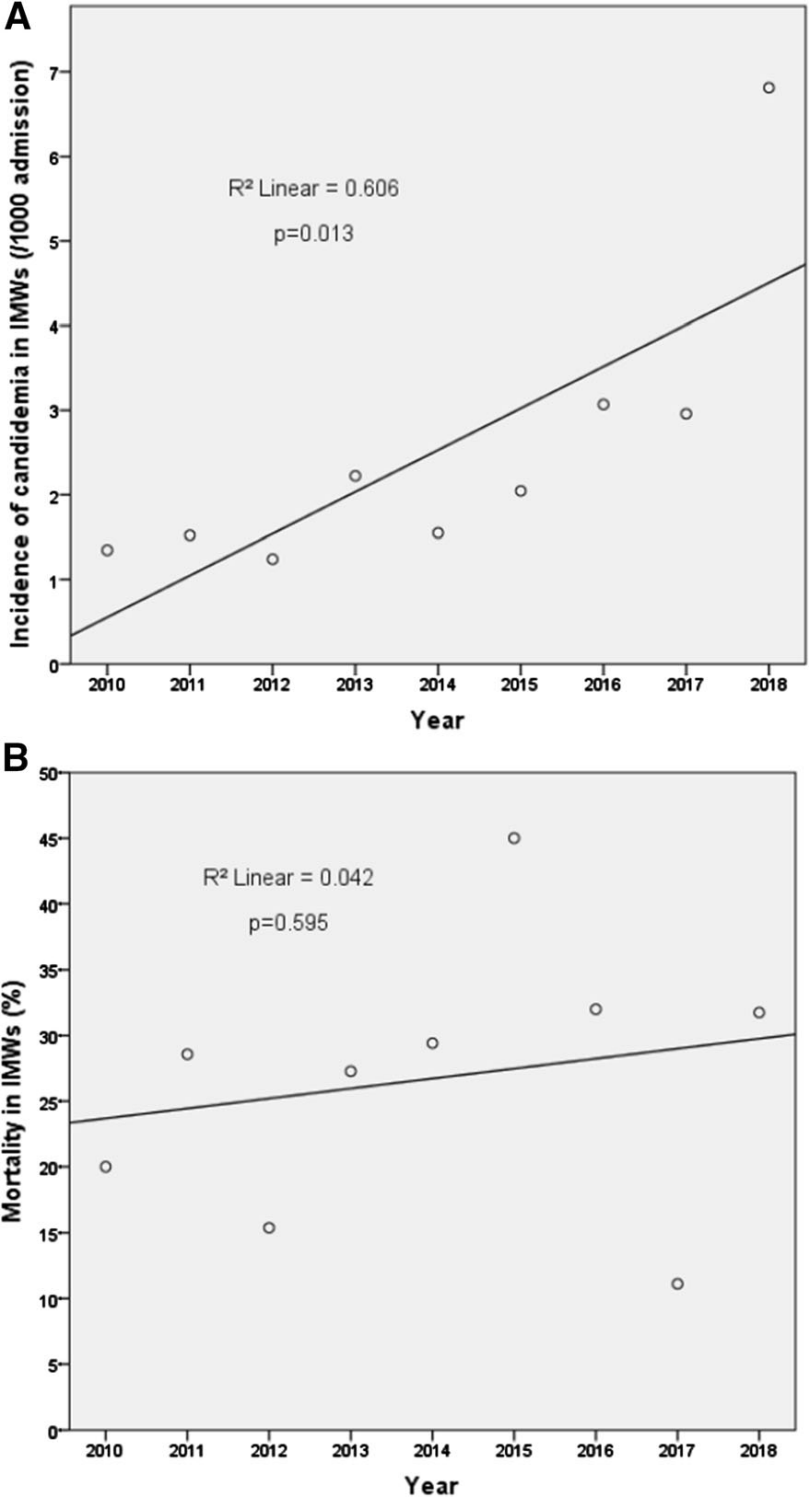
Incidence of candidemia (**a**) and mortality (**b**) in IMWs patients

During the study period, 505 cases of candidemia were diagnosed: 218 (43%) occurred in patients hospitalized in IMWs and 287 (57%) in patients admitted in other wards. The cumulative incidence of candidemia in IMWs was 2.5/1000 hospital admission, showing a significant increase over time (*p* = 0.013; Fig. 1a).

The baseline characteristics of the study population are reported in Table 1. Patients were predominantly male, with a median age of 68 years. Cardiovascular diseases and solid tumors were the most frequent comorbidities followed by gastrointestinal and neurological diseases. In most cases (78%), candidemia was considered CVC-related. *Candida albicans* accounted for 51% of the cases, followed by *C. parapsilosis* (25%), *C. tropicalis* (9%) and *C. glabrata* (7%).

**Table 1:** Characteristics of patients with candidemia hospitalized in Internal Medical Wards

Characteristics	All patients (n = 218)	Not surviving n = 60 (28%)	Surviving n = 158 (72%)	*p* value^a^
Male sex, *n (%)*	132 (60)	40 (18%)	92 (42%)	0.255
Age, median (IQR)^b^	68 (60–77)	71 (66–80)	66 (60–77)	** < 0.001**
*Chronic comorbidities*				
Chronic pulmonary diseases, *n (%) *^c^	24 (11%)	8 (13%)	16 (10%)	0.499
Hematological malignancy, *n (%)*	20 (9%)	10 (17%)	10 (6%)	**0.018**
Cardiovascular diseases, *n (%) *^d^	97 (45%)	29 (48%)	68 (43%)	0.482
Neurological diseases, *n (%) *^e^	57 (26%)	15 (25%)	42 (27%)	0.812
Gastrointestinal diseases, *n (%) *^f^	68 (31%)	11 (18%)	57 (36%)	**0.012**
Diabetes mellitus, *n (%)*	35 (16%)	13 (22%)	22 (14%)	0.164
Chronic renal failure, *n (%)*	28 (13%)	9 (15%)	19 (12%)	0.558
Solid tumors, *n (%)*	85 (39%)	27 (45%)	58 (37%)	0.268
Solid organ transplant, *n (%)*	11 (5%)	1 (2%)	10 (6%)	0.297
Surgery, *n (%)*	49 (23%)	13 (22%)	36 (23%)	0.860
Charlson’s score, median (IQR)	6 (5–7)	6 (5–7)	6 (4–7)	**0.007**
Central venous catheter, *n (%)*	185 (85%)	51 (85%)	134 (85%)	0.972
Central venous catheter-related BSIs, *n (%)*^g^	146 (78%)	42 (71%)	104 (66%)	0.489
Early central venous catheter removal, *n (%) *^h^	40 (18%)	6 (10%)	34 (22%)	**0.050**
Other devices, *n (%) *^i^	173 (79%)	49 (82%)	124 (79%)	0.604
Previous invasive procedures (< 72 h), *n (%) *^j^	47 (22%)	10 (17%)	37 (23%)	0.279
Parenteral nutrition, *n (%)*	144 (66%)	42 (70)	102 (65%)	0.448
Haemodialysis, *n (%)*	9 (4%)	2 (3%)	7 (4%)	0.716
Steroid therapy, *n (%)*	72 (33%)	20 (33%)	52 (33%)	0.953
Immunosuppressive therapy, *n (%)*	41 (19%)	14 (23%)	27 (17%)	0.292
Neutropenia, *n (%)*	11 (5%)	8 (13%)	3 (2%)	**0.002**
Pneumonia, *n (%)*	71 (33%)	30 (50%)	41 (26%)	**0.001**
Septic shock, *n (%)*	25 (13%)	13 (22%)	12 (7%)	**0.004**
Acute kidney failure, *n (%)*	14 (6%)	5 (8%)	9 (6%)	0.478
Concomitant bacteriemia, *n (%)*	112 (51%)	29 (48%)	83 (53%)	0.580
Pre-infection hospitalization, *median days (IQR)*	14 (7–34)	12 (7–34)	20 (7–35)	** < 0.001**
*Candida species*				
*Candida albicans, n (%)*	110 (51%)	39 (65%)	71 (45%)	**0.008**
*Candida parapsilosis, n (%)*	54 (25%)	8 (13%)	46 (29%)	**0.028**
*Candida tropicalis, n (%)*	20 (9%)	7 (12%)	13 (8%)	0.432
*Candida glabrata, n (%)*	16 (7%)	4 (7%)	12 (8%)	1
Other *Candida* species, *n (%) *^k^	16 (7%)	2 (3%)	14 (9%)	0.254
Appropriate antifungal therapy, *n (%) *^l^	124 (57%)	30 (50%)	94 (60%)	0.206
*Primary antifungal therapy*				
Azoles, *n (%)*	95 (54%)	21 (46%)	74 (56%)	0.204
Echinocandins, *n (%)*	78 (44%)	25 (54%)	53 (40%)	0.102
Polyenes, *n (%)*	4 (2%)	0	4 (3%)	0.573
No treatment, *n (%)*	41 (19%)	14 (23%)	27 (17%)	0.291

Bold letter means "significant" form a statistical point of view

^a^Categorical variables were compared by the χ2 or Fisher exact test, while continuous variables were evaluated by the Student *t* test or the Mann–Whitney *U* test

^b^IQR, Interquartile range

^c^Chronic pulmonary diseases include asthma, chronic bronchitis, emphysema and lung fibrosis

^d^Cardiovascular diseases include heart failure, ischemic heart disease, endocarditis and arrhythmia

^e^Neurological diseases include Parkinson’s disease, Alzheimer’s disease and paralysis

^f^Gastrointestinal diseases include Crohn’s disease, ulcerative colitis, chronic pancreatitis and gallbladder stones

^g^A catheter-related candidemia was defined according to the guidelines of the infectious diseases society of America [12]

^h^Early central venous catheter removal was considered occurring within 48 h from blood cultures drawing

^i^Other devices include urinary catheter, surgical drainage, cutaneous gastrostomy and tracheostomy tube

^j^Previous invasive procedures include endoscopy and positioning of any device

^k^Immunosuppressive therapy include calcineurin inhibitors and monoclonal antibodies

^l^Other *Candida* species included *Candida guilliermondii* (n = 5), *Candida lusitaniae* (n = 5), *Candida dubliniensis* (n = 2), and one isolate each of *Candida krusei*, *Candida kefyr*, *Candida pelliculosa* and *Candida rugosa*

^m^Appropriate antifungal therapy was considered when the appropriate drug with adequate dosage was started within 72 h the first blood culture performed

While over 80% of the patients (177/218) were treated with an antifungal agent, there were 19% of the population (41/218) who did not receive any antifungal treatment. Fluconazole was the most common drug utilized as primary therapy, followed by an echinocandin.

Crude mortality on day 30 from the onset of BSI was 28% (60/218; Table 1). The mortality rate did not show a significant increase over time (Fig. 1b). The following characteristics were significantly more common in patients with a negative outcome: older age, the presence of hematological malignancy, higher Charlson’s score, the presence of neutropenia, pneumonia, and septic shock, and being infected with *C. albicans* (*p*, ranging from < 0.001 to 0.018). On the opposite, the following characteristics were significantly more common in patients with positive outcome: the presence of gastrointestinal disease, longer pre-infection hospitalization, and being infected with *C. parapsilosis* (*p*, ranging from < 0.001 to 0.012). There was a trend, although not statistically significant (*p* = 0.05), of better outcome in patients with early CVC removal.

In the multivariate analysis, the presence of neutropenia, pneumonia and being infected with *C. albicans* emerged as independent predictors of mortality (Table 2).

**Table 2:** Fluconazole, caspofungin and amphotericin B susceptibility results for isolates belonging to the four most common *Candida* spp.^a^

*Candida* spp. (no. tested) / antifungal drug	MIC (µg/ml)			Resistant isolates (%)
	Range	MIC50	MIC90	
*Candida albicans (102)*				
Fluconazole	≤ 0.125– > 256	0.25	0.5	2
Caspofungin	≤ 0.008–0.5	0.03	0.125	0
Amphotericin B	≤ 0.125–1.0	0.5	1.0	0
*Candida parapsilosis (54)*				
Fluconazole	≤ 0.125–2.0	0.5	1.0	0
Caspofungin	0.03–1.0	0.5	1.0	0
Amphotericin B	0.125–1.0	0.5	1.0	0
*Candida tropicalis (20)*				
Fluconazole	0.5–4.0	1.0	2.0	0
Caspofungin	≤ 0.008–0.125	0.06	0.06	0
Amphotericin B	0.125–1.0	1.0	1.0	0
*Candida glabrata (16)*				
Fluconazole	2.0–32	8.0	32	0
Caspofungin	0.03–0.5	0.06	0.125	0
Amphotericin B	0.25–1.0	1.0	1.0	0

^a^MICs were interpreted according to latest species-specific clinical breakpoints as established by the Clinical and Laboratory Standards Institute (CLSI) [13]

**Table 3:** Risk factors associated with 30-day mortality in IMW patients with candidemia analyzed by logistic regression

Variable	OR	95% CI	*p*
Neutropenia	7,247	1,368–38,400	0.020
Pneumonia	2,323	1,105–4,884	0.026
*C.albicans*	2,642	1,223–5,708	0.013

Antifungal susceptibility results for the isolates belonging to the four most common *Candida* spp. are reported in Table 3. According to the CLSI breakpoints, 2% of the isolates of *C. albicans* were considered in vitro resistant to fluconazole. Resistance did not occurred for other isolate/antifungal combinations.

## Discussion

We demonstrated that the incidence of candidemia in patients admitted to IMWs is increasing. Our patient population showed to be fragile being affected by multiple comorbidities. The mortality rate was 28% on day 30. Overall, these figures are quite alarming indicating a new group of patients who are at risk of developing this life-threatening infection.

In this study, we showed that specific clinical and microbiologic factors were associated with a negative outcome. In particular, the presence of neutropenia and pneumonia increased the risk of death by approximately seven- and two-fold, respectively. The first variable can be easily explained by the fact that neutrophils are key participants in the defense against fungal infections [14]. We also can speculate that the occurrence of pneumonia, along with other clinical features (i.e., higher prevalence of cardiovascular diseases and solid tumors), worsens the overall clinical situation in a patient population in which the risk of death is already high. Data from the literature show that several underlying conditions in IMWs patients with candidemia might represent independent risk factors for mortality [4–9]. One study evaluated 274 patients and found that cirrhosis and neurologic diseases were independently associated with increased risk of death [15]. Other clinical conditions such as chronic obstructive pulmonary disease and chronic kidney failure were predictors of mortality [7, 16]. Finally, the presence of solid tumors has been linked to a poor prognosis [17].

Mortality rate has been rarely related to the species of *Candida*. Here, we found that being infected with *C. albicans* represented an independent risk of mortality. It must be noted that *C. albicans* possesses virulence traits, such as the ability of transition from blastospore to hyphae, the presence of adhesins and the secretion of hydrolytic enzymes which make it more pathogenic than other species [18]. Furthermore, the amount of biofilm produced by isolates of *C. albicans* is generally higher than those produced by other species including the new emerging MDR yeast *C. auris* [19]. There are reports indicating as IMWs patients with candidemia due to *C. tropicalis* have a significantly higher mortality rate than patients infected with other species [7, 20]. It is interesting to note that *C. tropicalis* possesses well characterized virulence factors which resemble those of *C. albicans* [21].

Interestingly, we found that resistance was uncommon among isolates of *Candida* spp. causing infections in our patients. Only 2% of *C. albicans* isolates showed in vitro resistance to fluconazole. These data are in line with that recently observed in the literature. One international study investigated the in vitro activities of fluconazole and echinocandins against over 15,000 clinical isolates of *Candida* spp. and found that the overall resistance rate was rare. However, this study showed that a slow and steady emergence of resistance to both antifungal classes was observed in *C. glabrata* and *C. tropicalis* isolates [22].

Although our study was not designed to compare antifungal regimens, we observed that the choice of drug utilized as primary therapy (i.e.: fluconazole *vs* echinocandin) did not influence the outcome. Literature data report conflicting results on this issue [7, 15, 23–26]. A multicenter study investigated the role of antifungal therapy in the outcome of *C. glabrata* bloodstream infections and found that initial fluconazole treatment, a therapeutic drug regimen which might be suboptimal for infections caused by this species, was not associated with a poorer outcome than that obtained with echinocandins or L-AmB regimens [23]. Similarly, one regional italian study involving 230 candidemic patiens admitted to IMWs, showed that the type of antifungal treatment did not influence the outcome [7]. On the contrary, one recent study investigated the clinical characteristics, management and outcome of 111 patients with invasive candidiasis hospitalized in IMWs and found that fluconazole as initial therapy was associated with an increased risk of death at 90 days [24]. Overall, these data further underline the discrepancy between the clinical practice and the results of this studies. Indeed these studies, as well the international guidelines, indicate the superiority of echinocandins for the primary treatment of invasive candidiasis [25, 26].

The finding that 19% of our population did not undergo any therapy is worrying. This data has also been reported in other studies [7, 27]. This fact may be due to delayed diagnosis and to the rapid clinical evolution of patients. Another plausible reason is the efficiency of the antifungal stewardship in a given hospital/medical department. It has been recently demonstrated that the lack of any antifungal regimen in candidemic patients admitted to IMWs range from 6 to 12% and from 17 to 25% in hospitals where the infectious disease consultant is available or unaivalable, respectively [7]. It is intriguing to note that the lack of any antifungal therapy did not affect the outcome. Although we observed a higher proportion of death in untreated *vs* treated patients (23% *vs* 17%), the difference was not significant. Whether these unexpected results were due to the fact that untreated patients were less clinically compromised or better managed in controlling the source of infection was not investigated.

Our study have several limitations: it is a single‐center retrospective observational study; due to the absence of a control group we couldn’t make any causality inference in this setting; furthermore, some patients were not screened for secondary locations, so complications may have been underestimated.

In conclusion, we showed that patients admitted to IMWs are increasingly at higher risk of developing candidemia. Mortality rate remains high and significantly associated with both microbiologic- and host-related factors. Further prospective studies are strongly encouraged to investigate risk factors for development and management of candidemia in patients admitted to IMWs.

## Data Availability

The datasets used and/or analyzed during the current study are available from the corresponding author on request.
